# A Space Telescope Scheduling Approach Combining Observation Priority Coding with Problem Decomposition Strategies

**DOI:** 10.3390/biomimetics9120718

**Published:** 2024-11-21

**Authors:** Kaiyuan Zhang, Bao-Lin Ye, Xiaoyun Xia, Zijia Wang, Xianchao Zhang, Hai Jiang

**Affiliations:** 1 School of Information Science and Engineering, Jiaxing University, Jiaxing 314001, China; kaiyuanz0101@163.com (K.Z.); xiaxiaoyun@zjxu.edu.cn (X.X.); 2 School of Science, Jiangxi University of Science and Technology, Ganzhou 341000, China; 3 School of Computer Science and Cyber Engineering, Guangzhou University, Guangzhou 510006, China; 4 Institute of Information Network and Artificial Intelligence, Jiaxing University, Jiaxing 314001, China; zhxianchao@zjxu.edu.cn; 5 National Astronomical Observatories, Chinese Academy of Sciences, Beijing 100012, China; jhai@nao.cas.cn

**Keywords:** telescope observation scheduling, coding method, decoding method, neighborhood search, combinatorial optimization

## Abstract

With the increasing number of space debris, the demand for telescopes to observe space debris is also constantly increasing. The telescope observation scheduling problem requires algorithms to schedule telescopes to maximize observation value within the visible time constraints of space debris, especially when dealing with large-scale problems. This paper proposes a practical heuristic algorithm to solve the telescope observation of space debris scheduling problem. In order to accelerate the solving speed of algorithms on large-scale problems, this paper combines the characteristics of the problem and partitions the large-scale problem into multiple sub-problems according to the observation time. In each sub-problem, a coding method based on the priority of the target going into the queue is proposed in combination with the actual observation data, and a decoding method matching the coding method is designed. In the solution process for each sub-problem, an adaptive variable neighborhood search is used to solve the space debris observation plan. When solving all sub-problems is completed, the observation plans obtained on all sub-problems are combined to obtain the observation plan of the original problem.

## 1. Introduction

The space debris monitoring network regularly tracks and catalogs over 28,000 pieces of debris, and the total mass of artificial objects in low Earth orbit exceeds 9200 tons [1,2,3]. Given the harm of space debris to the operational safety of spacecraft and the potential impact threat of near-Earth objects on Earth, the demand for space debris detection and early warning in various countries is constantly increasing [4,5]. Space observation equipment is being constructed in various countries worldwide to meet space observation needs [6,7,8]. In 2016, the Five-hundred-meter Aperture Spherical Radio Telescope (FAST), the world’s largest and most sensitive single-aperture radio telescope built independently by China, was fully completed and put into operation. The scheduling of FAST requires maximizing the number of observable proposals and the overall scientific priority and minimizing the slew cost generated by telescope shifting while considering the constraints. The researchers modeled the problem as a minimum-cost maximum-flow problem, designed a method based on maximum matching edge detection to reduce the problem size, and proposed a backtracking algorithm to minimize the transformation cost of the optimal scheduling [9,10,11,12]. Compared with individual telescopes, telescope arrays allow for much greater observational accuracy and range by synchronizing multiple telescopes.

Researchers have done a great deal of research on the telescope scheduling algorithm to meet the needs of the observing equipment. The Gravitational Wave Optical Transient Observer prototype instrument (GOTO) was inaugurated in July 2017 in La Palma, Canary Islands [13,14]. GOTO’s telescope scheduling system consists of several independent control processes, with a master named ‘pilot’ overseeing the other processes. Observations are determined by an instantaneous scheduler instructing the ‘pilot’ on the targets to observe in real time and providing fast follow-up of transient events [13]. The scheduler for the GLORIA telescope network was designed with three algorithms to maximize the total network acceptance rate and minimize the time elapsed between observation submissions and results [15]. The first algorithm is based on the weather forecasting of the telescope position, the second algorithm is based on fuzzy logic using different input parameters, and the third algorithm is based on predicting the conditional probability that each telescope will receive an observation. After that, GLORIA researchers explore new machine learning methods, such as neural networks, support vector machines, etc., comparing these methods with the three algorithms mentioned above [15]. A prediction-type scheduler is used in Algeria’s National Aures Observatory (NAO), which solves the scheduling problem using a multi-objective genetic algorithm (named NSGA-II) based on Pareto optimality [16]. The INO340 telescope of the Iranian National Observatory aims to minimize the idle time of the telescope and reduce its mechanical motion costs while obtaining the best quality image results [17]. INO340 uses genetic algorithms to consider predictable factors that affect observation conditions and obtain the optimal scheduling plan [17]. In 2023, Zhang et al. proposed a multilevel scheduling model for the time-domain survey telescope array scheduling problem. They encapsulated the functionality in software with a hierarchical architecture, developing a flexible framework and proposing an optimal metric to maintain uniform coverage and efficient time utilization from a global perspective [18].

The current research has fewer concerns about reducing the dimensionality and improving the solution efficiency for large-scale telescope scheduling problems. However, the above problems often exist in the scheduling problems of the actual telescope observation of space debris. In this paper, we design a problem decomposition strategy to reduce the dimensionality and solution time of the problem according to the problem characteristics. By drawing on similar job shop scheduling problems, mutually cooperative coding and decoding methods are proposed to enhance problem-solving efficiency. The performance of the algorithm and the proposed strategy are verified using adaptive variable domain search in conjunction with the above strategy on real-world arithmetic cases provided by the Observatory.

## 2. Telescope Observation Scheduling Problem

Telescope observation of space debris is a typical practical scheduling problem, where it is necessary to determine which telescope will observe which space target at what time before telescope observation. The role of algorithmic scheduling in the process of telescope observation of space debris is crucial, and this has attracted a large number of researchers to carry out related research work. The model proposed in this paper is as follows.

We suppose there are *N* targets in space. For any target n∈{1,…,N}, each target is assigned a priority level $l_{n}$, where $l_{n}\in \left\{1,\ldots ,R\right\}$ and *R* is a constant (in this article, it is 9) representing its importance. These levels are used to rank and allocate telescope resources among the N independent targets. The bigger the number of observation levels of the target the higher the observation value. Each target can be observed only within the time window, denoted as $\left[O_{n},D_{n}\right]$, and $D_{n}-O_{n}\geq T_{n}$. $O_{n}$ is the initial time when the target can be observed, $D_{n}$ is the deadline, and $T_{n}$ is the time taken by the telescope to observe the target *n*. This paper assumes that the time $T_{n}$ required for a telescope to observe any target is 90 s.

**Figure 1:**
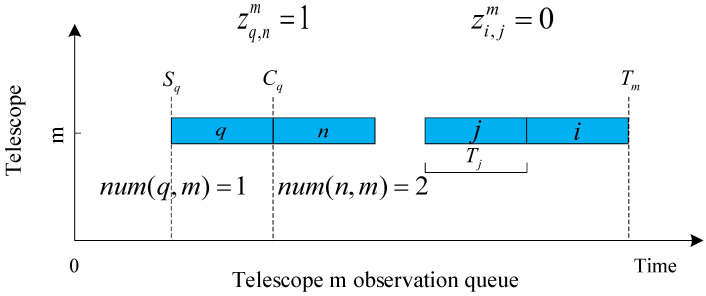
Telescope m’s observation queue Gantt chart.

Let there be a total of *M* telescopes on the ground and any telescope m∈{1,…,M}. A telescope can observe only one target at a time, and each target can be assigned to only one telescope for observation. When a telescope starts observing a target, it needs to observe the target for 90 s before it can observe the next target, with the switching time between telescope targets being ignored. When a target has already been observed, all telescopes will not observe it again. We define a Boolean variable $x_{n,m}$, where $x_{n,m}=1$ to indicate that target *n* is in the observation queue of telescope *m*, and $x_{n,m}=0$ to indicate that target *n* is not in the observation queue of telescope *m*. $S_{n}$ and $C_{n}$ are the start and end observation times of target *n* in the telescope observation queue. We assume that *q* is a target of {1,…,N} and n≠q. $C_{q}$ is the observation end time of *q*. The calculation formulas for $S_{n}$ and $C_{n}$ are as follows:

(1) $$S_{n}=\left\{\begin{array}{c} O_{n},if\;\sum_{i=1}^{N}z_{i,n}^{m}=0,\;x_{n,m}=1\\ \max\left(O_{n},C_{q}\right),if\;z_{q,n}^{m}=1,\;x_{n,m}=1,num\left(n,m\right)-num\left(q,m\right)=1\\ \max\left(O_{n},T_{m}\right),if\;\sum_{j=1}^{N}z_{n,j}^{m}=0,\;x_{n,m}=1 \end{array}\right.$$

(2) $$C_{n}=S_{n}+T_{n}$$

where $z_{q,n}^{m}$ is a Boolean variable, and $z_{q,n}^{m}=1$ is denoted by the fact that both target *q* and target *n* are in the observation queue of telescope *m*, and *q* precedes *n*. In all other cases $z_{q,n}^{m}=0$. $num\left(n,m\right)$ denotes the observing order when target *n* is inserted into the current observing queue of telescope *m*. The meaning of num(n,m)−num(q,m)=1 is that both target *q* and target *n* are in the current observation queue of telescope *m*, and target *q* is the previous neighboring target of target *n* at the position to be inserted in the current observation queue of telescope *m*. $z_{q,n}^{m}$ and num(n,m) are shown in Figure 1. The horizontal axis in the figure is the time axis, representing the starting and ending observation times of the target in the telescope observation queue. The vertical axis represents the telescope number, indicating that targets *q*, *n*, *i*, and *j* are in the observation queue of telescope *m*. $S_{q}$ represents the start observation time of target *q*, and $C_{q}$ represents the end observation time of target *q*. $T_{j}$ is the observation time of target *j* in the telescope queue, and $T_{m}$ represents the end observation time of the observation queue for telescope *m*. Due to *q* preceding *n*, $z_{q,n}^{m}=1$ and $z_{n,q}^{m}=0$. In addition, *q* is the first target observed in the observation queue of telescope *m*, so $num\left(q,m\right)=1$, and, similarly, $num\left(n,m\right)=2$. We define $T_{m}$ as the end observation time of the last target in the observation queue of telescope *m*, which updates with the change of the observation queue. When there is no target in telescope *m*’s queue, we set the $T_{m}$ value to 0.

As shown in Equation (1), the values of $S_{n}$ are divided into three cases:

1. The target is inserted at the head of the observing queue. Target *n* is added to the current observation queue of telescope *m* as the head observation target (see Figure 6 in Section 3.3.2 for details). At this point, target *n* is at the head of the queue, so, for any target *i* (regardless of whether it is in the queue of *m*), i∈{1,…,N},i≠n, there is $z_{i,n}^{m}=0$, i.e., $\sum_{i=1}^{N}z_{i,n}^{m}=0,\;x_{n,m}=1$. To simplify the experiment and select $S_{n}$ as early as possible, let $S_{n}=O_{n}$.
2. The target is inserted into the middle of the observation queue. Target *n* is inserted into the middle of the observation queue of telescope *m*. At this point, target *q* is the previous target adjacent to the insertion position of target *n* (see Figure 7 in Section 3.3.2 for details), i.e., $z_{q,n}^{m}=1$, $x_{n,m}=1$ and num(n,m)−num(q,m)=1. We simplify the experiment to obtain the value of $S_{n}$ as early as possible while satisfying the constraints; then, the value of $S_{n}$ is $\max(O_{n},C_{q})$.
3. The target is inserted at the end of the observing queue. The target *n* is added to the current observing queue of telescope *m* as the tail of the queue (see Figure 8 in Section 3.3.2 for details); so, for any target *j* (regardless of whether it is in the queue of *m*), j∈{1,…,N},j≠n, there is $z_{n,j}^{m}=0$, i.e., $\sum_{j=1}^{N}z_{n,j}^{m}=0,x_{n,m}=1$. The value of $S_{n}$ is $\max(O_{n},T_{m})$.

Equation (2) represents the calculation method for the end observation time $C_{n}$. After determining $S_{n}$, $T_{n}$ is set to 90s, i.e., $C_{n}=S_{n}+T_{n}$. If the target is inserted into the tail of the observation queue, $T_{n}$ also needs to be updated, that is, $T_{m}=C_{n}$.

It should be noted that, in the insertion method designed in this article, every time a new target is inserted into the telescope observation queue, a free time window that meets the constraints in the observation queue will be selected (without affecting the start and end observation times of other targets that have already entered the observation queue). The start observation time should be set as early as possible. If the target does not have a free window time in the queue that meets the constraints, the target will not be inserted into the observation queue of the current telescope.

In order to observe as many high-observation-value targets as possible, the telescope scheduling goal is the total target successful observation value *L*, denoted as

(3) $$L=\sum_{n=1}^{N}\sum_{m=1}^{M}x_{n,m}\cdot l_{n}$$

**Figure 2:**
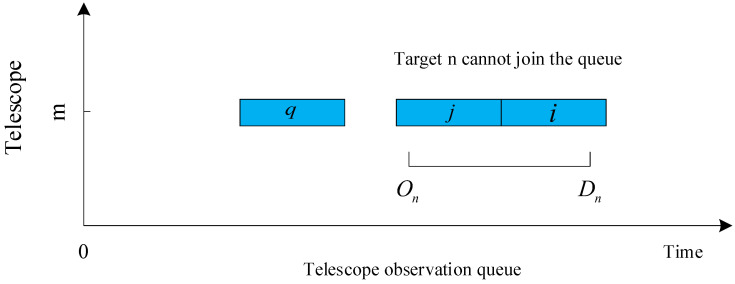
Schematic of observation queue time constraints.

The telescope scheduling model is established as follows:

(4) $$\max L=\sum_{n=1}^{N}\sum_{m=1}^{M}x_{n,m}\cdot l_{n}$$

(5) $$C_{n}\leq S_{q}+z_{q,n}^{m}\cdot Y$$

(6) $$C_{q}\leq S_{n}+z_{n,q}^{m}\cdot Y$$

(7) $$z_{n,q}^{m}+z_{q,n}^{m}=1\cdot Y$$

(8) $$\sum_{m=1}^{M}x_{n,m}\leq 1$$

(9) $$C_{n}=\left(S_{n}+T_{n}\right)$$

(10) $$S_{n}\geq O_{n}$$

(11) $$D_{n}\geq C_{n}$$

(12) $$x_{n,m}=\left\{0,1\right\}$$

(13) $$z_{n,q}^{m}=\left\{0,1\right\},z_{q,n}^{m}=\left\{0,1\right\}$$

n∈{1,…,N}, q∈{1,…,N}, n≠q, $l_{n}\in \{1,\ldots ,R\}$, and m∈{1,…,M}. Constraints (5), (6), and (7) indicate that a telescope can observe only one target at a time, as shown in Figure 2. Targets *q*, *j*, and *i* are in the observation queue of telescope *m*. Since the observation time of targets *i* and *j* in the queue coincide with the observable period of *n*, and the telescope can only observe one target at a time, *n* cannot join the observation queue of *m*.

Among them, *Y* is a large positive number. For the constraint (5), if $z_{q,n}^{m}=1$, the constraint loses its restrictiveness; for the constraint (6), if $z_{n,q}^{m}=1$, the constraint loses its restrictiveness.

The constraint (8) means that each target can only be assigned at most one telescope.

The constraint (9) represents the value of the observation end time $C_{n}$ of target *n*.

The constraint (10) indicates that the observation start time must be greater than or equal to the initial time when the target can be observed. Constraint (11) states that the observation end time must be less than the observable deadline of the target.

The constraints (12) and (13) represent $x_{n,m}$, and $z_{n,q}$ are Boolean variables.

The established model aims to find the optimal scheduling policy $\sigma^{*}=\left\{x_{n,m}^{*},S_{n}^{*}\right\}$, (n∈{1,…,N}, m∈{1,…,M}) to maximize the total target observation value.

The simplified data of some observation targets are shown in Table 1.

In Table 1, each target has its unique corresponding target number *n*. In an instance, each target has at least one observable period (from initial time to deadline) and may have multiple observable periods. For example, Target 16 has three observable periods. Not every target can ultimately be observed. For each target’s observable period, the target will not be observed if no available time slot matching this period can be found in the observation schedules of all telescopes. The target level $l_{n}$ represents the target’s observation level; the bigger the target’s observation level value, the higher the observation value. $O_{n}$ is the initial time of the observable period for target *n*, and $D_{n}$ is the deadline of the observable period for target *n*, measured in seconds. To simplify the experiment, the observation window for each target in the article is the same for each telescope.

## 3. A Heuristic Approach Combining Problem Decomposition

### 3.1. Algorithm’s Overall Flow

According to the particularity of the telescope observation scheduling problem, the original problem can be divided into multiple sub-problems for solution. The observation queues obtained by solving the divided sub-problems do not overlap in time. After solving the sub-problems separately, the observation plans obtained can be combined to obtain the observation plan of the original problem.

**Figure 3:**
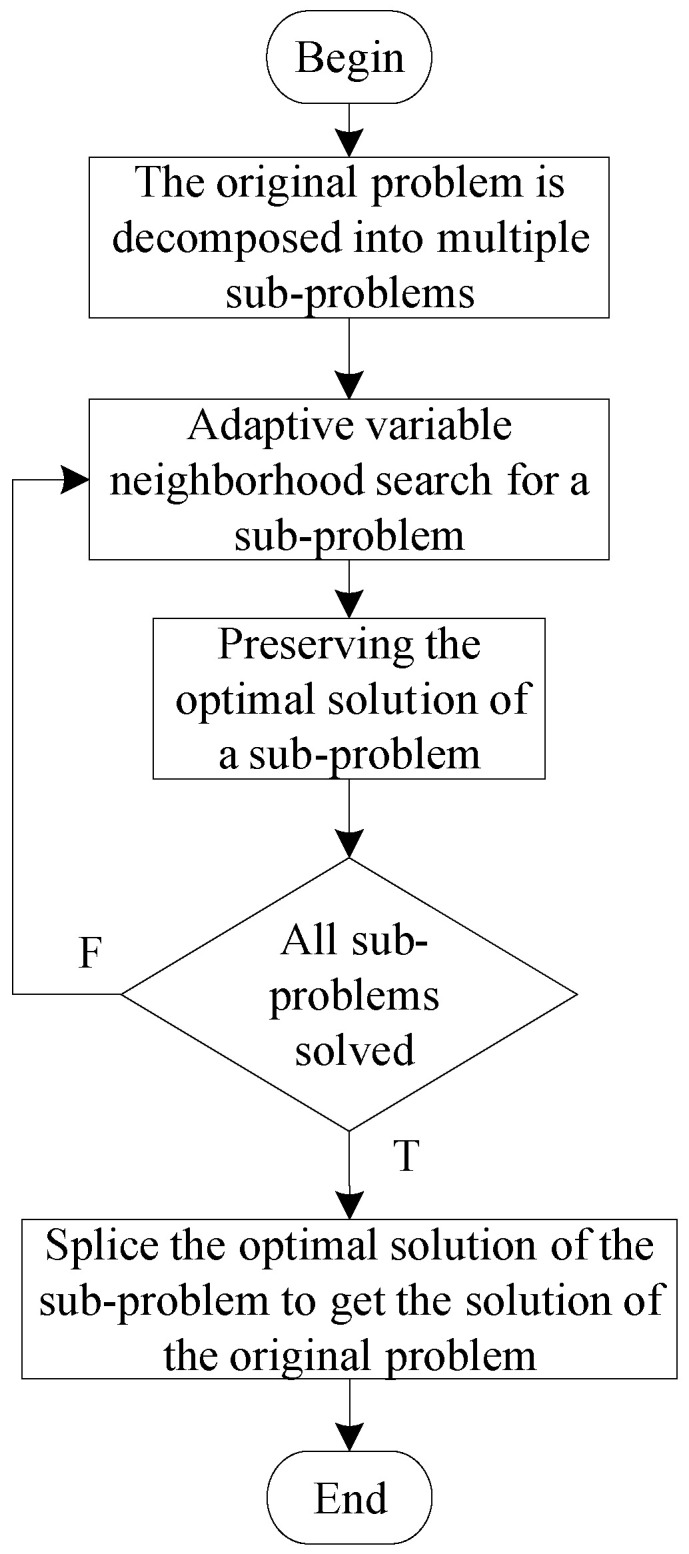
Algorithm’s overall flow chart.

The original problem can be divided into sub-problems recursively. After using the problem decomposition strategy, the algorithm flow chart is shown in Figure 3.

The algorithm’s overall execution process is as follows:

1. A sub-problem is solved to obtain an observation plan for that sub-problem. If the space debris in that observation plan appears again in other unsolved sub-problems, it is removed from the unsolved sub-problems.
2. Repeat the above steps until all sub-problems are solved and corresponding observation plans are obtained.
3. The observation plan obtained by solving all sub-problems is combined to obtain the observation plan for the original problem.

### 3.2. Problem Decomposition Strategy

**Figure 4:**
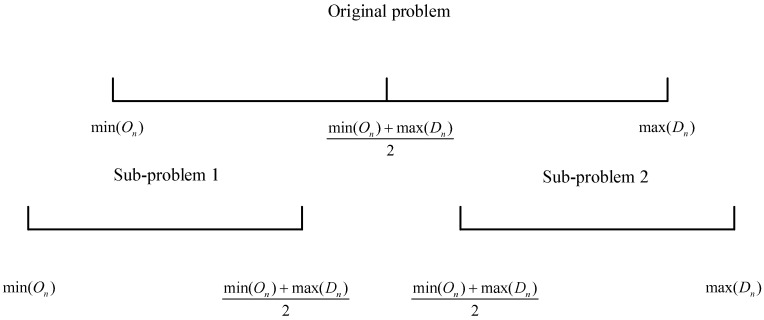
Schematic diagram of problem decomposition.

The observation target dimension of the telescope observation scheduling problem is enormous, and, on average, the observation scheduling of thousands of space targets needs to be processed daily. In order to reduce the dimension of the problem, a problem decomposition method is proposed based on the characteristics of the problem in this article:

1. Sort all target data in ascending order according to $O_{n}$, as shown in Table 2.
2. After sorting, add the minimum value of $O_{n}$ and the maximum value of $D_{n}$ in all data and divide by 2 as the segmented time point, as shown in Figure 4.
3. The data with $O_{n}$ and $D_{n}$ less than the segmented time point are added to the set of the first sub-problem.
4. The data with $O_{n}$ and $D_{n}$ greater than the segmented time point are added to the set of the second sub-problem.
5. If the segmented time point is greater than $O_{n}$ and less than $D_{n}$, the observable period of the target is divided into two periods. One is from $O_{n}$ to the segment time point, and the other is from the segment time point to $D_{n}$. If the length of the time period after separation is less than $T_{n}$, it will be discarded. Otherwise, the previous time segment is added to the first sub-problem. The following data segment is added to the set of the second sub-problem.

Taking the data in Table 2 as an example, the minimum value of $O_{n}$ is 77,539 in the data of target 2378, and the maximum value of $D_{n}$ is 80,267 in the data of target 2121. We add 77,539 to 80,267 and divide by 2 to obtain the segmented time point 78,903.

The observation period for target 2378 is divided into two segments, 77,539 to 78,903 and 78,903 to 79,309. Both segments have a range greater than 90 s. The data for the first segment are added to sub-problem 1, and the data for the second segment are added to sub-problem 2.

The $O_{n}$ and $D_{n}$ of targets 6848, 2377, and 4259 are all less than 78,903, and the data are directly added to the set of sub-problem 1. The $O_{n}$ and $D_{n}$ of targets 4617, 6266, and 2121 are all greater than 78,903, and the data are directly added to the set of sub-problem 2. The data of the two sub-problems obtained after problem decomposition are shown in Table 3 and Table 4.

The observation plan based on the data in Table 2 on a telescope is shown in Table 5. The observation plan obtained from Table 3 and Table 4 is also the same as the observation plan in Table 5.

It is demonstrated through experiments in the subsequent chapters that the use of the problem decomposition strategy significantly reduces the algorithm solution time when compared to the case where the solution is done directly without using the problem decomposition strategy.

It is worth mentioning that the experimental results in subsequent chapters show that using the problem decomposition strategy to decompose the problem and reduce its dimensionality each time continuously saves less and less computational time. At the same time, the observation plan obtained by using a small number of times of problem decomposition is better than the one obtained by using multiple times of problem decomposition. In the case where the 1000 pieces of data are taken from each of the two two-telescope examples, the observation plan obtained by decomposing the original problem into four sub-problems is better than that obtained by decomposing the original problem into two sub-problems. Taking instance 1 as an example, the period of the original problem is 74,143 to 113,650. After decomposing into four sub-problems, the period of sub-problem 1 is 74,143 to 84,019, the period of sub-problem 2 is 84,019 to 93,896, the period of sub-problem 3 is 93,896 to 103,565, and the period of sub-problem 4 is 103,565 to 113,650.

### 3.3. Coding and Decoding

The main factors affecting the scheduling of telescope observations in the problem studied in this paper are as follows [19]:

1. Level of observation targets.
2. Target observable time window.
3. Observation time of the telescope on the target.

The problem has many constraints, and the dimension of the solution space of the problem is vast, necessitating the minimization of the solution space as much as possible, and, at the same time, requires the efficiency of the coding operation, which is the basis for the subsequent algorithms to be able to solve the problem efficiently. Designing a reasonable decoding method with appropriate coding to reduce the solution space size is the focus of the research problem.

#### 3.3.1. Coding Method

In order to solve the above problems, this paper uses the target observation data provided by the observatory to propose a coding method based on the priority of the target entering the telescope observation queue.

This paper takes the random initial solution $\left[58,12,29,16,13\right]$ of the problem obtained from the data in Table 1 as an example. Each variable in the solution indicates the number of the observation target, and the earlier the target appears in the solution, the earlier it is added to telescope observation by the decoding method. According to the target entry priority in the above solution, the sorted data to be added to the observation queue are obtained, as shown in Table 6.

Taking Table 6 as an example, the coding method in this paper reduces the problem size of 8 to the solution coding of five decision variables, which reduces the solution space and also facilitates the operators to operate efficiently on the solution, which lays the foundation for the subsequent algorithms to solve the problem efficiently. Which telescope the subsequent target is added to and when it is added are handled by a decoding method that matches the coding method. The coding part is only responsible for generating a data table sorted according to priority based on the target priority in the solution, and the decoding method reads the data table to decide which telescope the target joins and when it joins the telescope.

#### 3.3.2. Decoding Method

**Figure 5:**
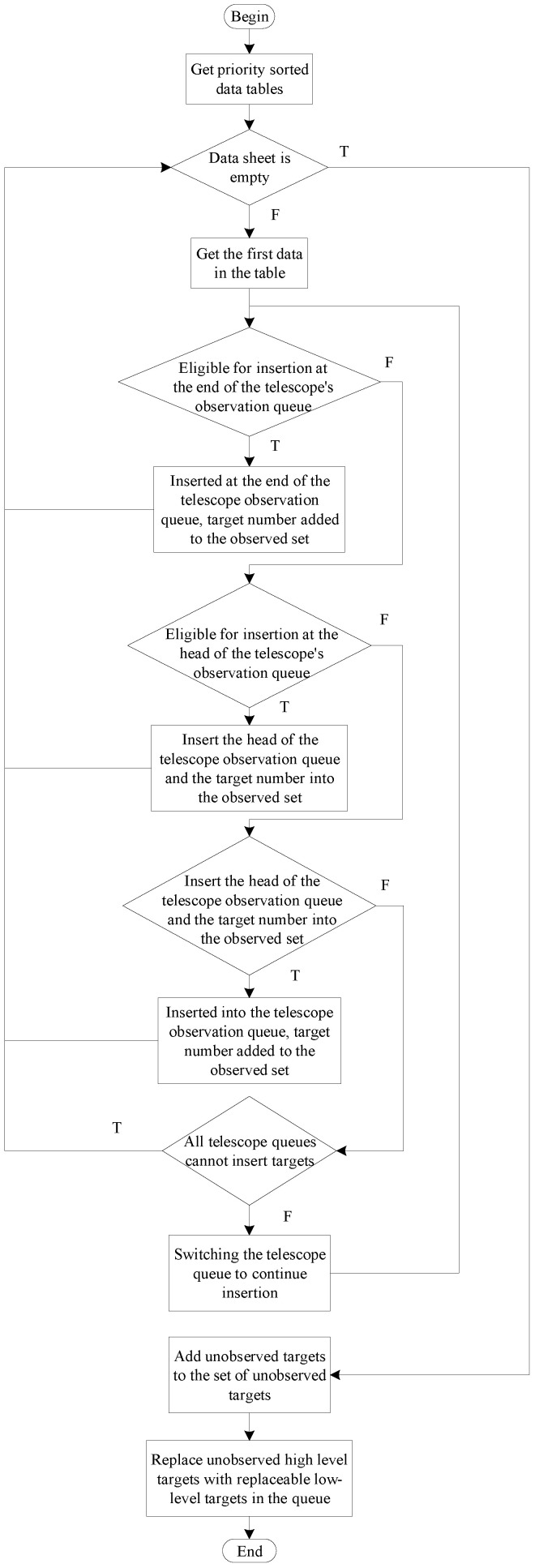
Flow chart of proposed decoding method.

Applying the decoding approach has achieved significant results in similar scheduling problems (job shop scheduling) and, together with the appropriate coding approach, it can significantly reduce the search space [20,21]. The essence of the algorithm operation solution in the telescope scheduling problem is to allow as many targets as possible to join the observation queue of the telescope while selecting high observation value targets as much as possible to join the observation queue. Based on this property of the telescope scheduling problem, this paper proposes a decoding method that matches the abovementioned coding method. The flowchart of the decoding method is shown in Figure 5.

**Figure 6:**
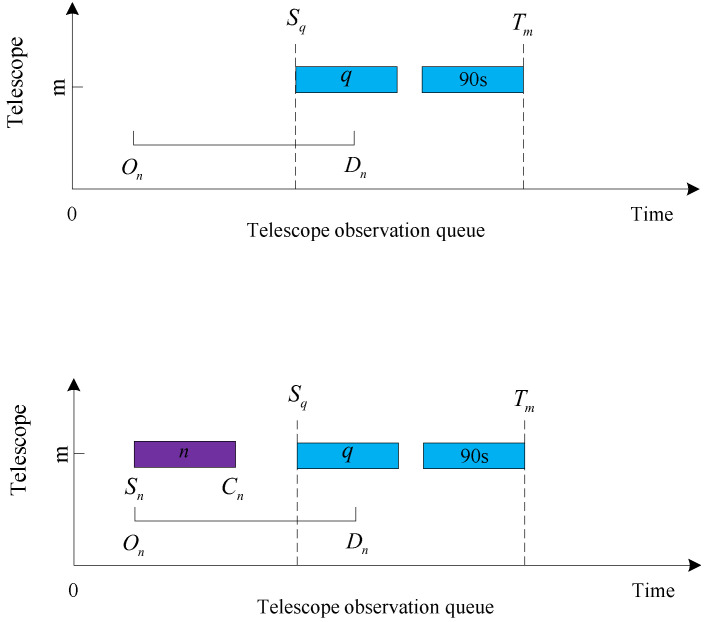
Queue head insertion.

**Figure 7:**
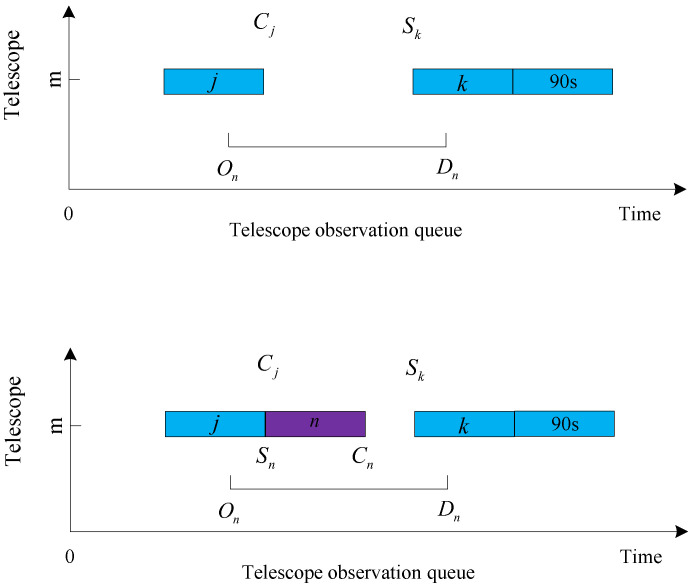
Insertion in the middle of the queue.

**Figure 8:**
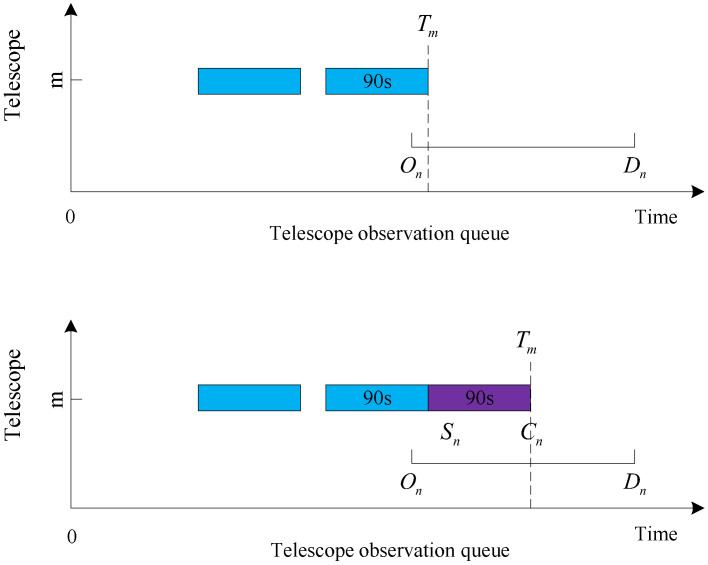
Insertion at the end of the queue.

**Figure 9:**
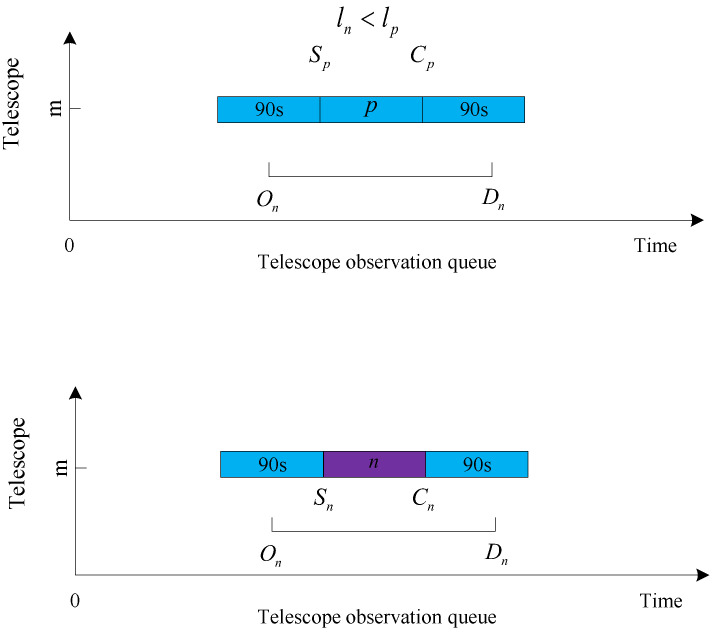
Replacement.

The judgment conditions for the four cases are given first:

1. Queue head insertion condition: the target was not observed, and $S_{q}-O_{n}\geq T_{n}$, where *q* is the first target in the observation queue of the current telescope *m*, and the observation start time is $S_{q}$. The queue head insertion is shown in Figure 6.
2. Insertion condition in the middle of the queue: $S_{k}-C_{j}\geq T_{n}$ and $S_{k}-O_{n}\geq T_{n}$ are satisfied simultaneously, and the target is not observed, where *j* is the last neighboring observed target at the location to be inserted, and *k* is the next neighboring observed target at the location to be inserted. The team insertion is shown in Figure 7.
3. Insertion conditions at the end of the queue: the target is not observed, and $D_{n}-T_{m}\geq T_{n}$. The end of the queue is inserted, as shown in Figure 8.
4. Replacement condition: the target is not observed while satisfying $l_{n}<l_{p}$, $O_{n}\leq S_{p}$ and $C_{p}\leq D_{n}$, where *p* is the observed target in the current queue. The replacement insertion is shown in Figure 9.

It is worth mentioning that, after a large number of repeated experiments, it was found that the observation queue obtained by the decoding method is only related to the order in which the target enters the queue, and is not related to the order in which the three insertion methods are used.

The execution steps of the decoding method are as follows:

1. Obtain the first data in the list of observed target data.
2. Determine whether the acquired target data can be added to the first telescope observation queue. Judge whether the target can be added to a telescope queue in order to perform the following four steps: (A) Determine whether the target can be inserted into the tail of the observation queue, and if the insertion conditions are met, insert it into the tail of the observation queue. (B) Determine whether the target can be inserted into the observation queue head, and if the insertion conditions are met, insert it into the observation queue head. (C) Determine whether the target can be inserted into the middle of the observation queue, and if the insertion criteria are met, insert it into the middle of the observation queue. (D) If the target is inserted into the current telescope observation queue, the target number will be added to the collection of observed queues; otherwise, continue to judge whether the target can be added to the observation queue of the second telescope until the target is inserted into the telescope queue or each telescope queue can not be inserted, the end of the insertion process.
3. After completing step 2 above, regardless of whether the target is added to the observation queue or cannot be added to the observation queue, read the target data in the following data table and continue with step 2 until all data have been executed in step 2.
4. After all target data in the data table have been inserted and judged, unobserved targets are added to the unobserved target set.
5. Determine whether each unobserved target can replace the observed low-value target. If the conditions are met, the low-value target will be removed from the observation queue, and the high-value target will occupy the position of the removed low-value target in the observation queue. Finally, the scheduling ends after all unobserved targets have executed the replacement judgment.

Multiple replacement rounds were attempted in the experiment, testing the strategy mentioned in Step 5 until no low-value targets could be replaced in the telescope queues. This improved the total observation value but significantly increased the algorithm’s computation time. Therefore, fewer replacement rounds strike a better balance between total observation value and algorithm-solving time.

For the convenience of setting up only one telescope, the solution $\left[58,12,29,16,13\right]$ in Section 3.3.1 uses the decoding method proposed in this article to obtain the observation queue formation process, as shown in Table 7 and  Table 8.

Table 7 shows the observation queue after the insertion of all data tables. The process by which targets enter the observation queue is as follows:

1. Target 58 is the first to enter the observation queue, $S_{n}$ is 76,925, and $C_{n}$ is 77,015.
2. Target 12 does not fulfill the conditions to enter the observation queue.
3. Target 29 enters the end of the observation queue with an $S_{n}$ of 106,755 and a $C_{n}$ of 106,845.
4. Target 16 enters the observation queue header with an $S_{n}$ of 75,833 and a $C_{n}$ of 75,923.
5. Target 13 enters the observation queue queue with an $S_{n}$ of 77,015 and a $C_{n}$ of 77,105.

Table 8 shows the observation queue after performing the replacement operation. The observed value of the unobserved target 12 is higher than the replaceable observed target 58, executing the replacement operation.

### 3.4. Adaptive Variable Neighborhood Search Algorithm

Adaptive variable neighborhood search (AVNS) has achieved better results in many huge neighborhood problems [22,23,24,25,26,27,28]. By combining several operators to dynamically compete for neighborhood search opportunities, adaptive variable neighborhood search has good adaptability and scalability [29,30,31]. This paper’s adaptive variable neighborhood search uses the insertion, commutative, and two-opt operators. The operator dynamic competition strategy from the literature [24] is adopted, and the dynamic competition strategy is as follows: the insertion operator, the commutative operator, and the two-opt operator are set with weights $W_{1}$, $W_{2}$, and $W_{3}$, respectively, and the initial value of $W_{1}$, $W_{2}$, and $W_{3}$ is 1.0. When an operator searches for a better solution, its weight increases by 1.0. If an operator fails to find a better solution, the weight of this operator remains unchanged, while the weight of other operators increases by 0.5. The *W* update formula is as follows:

(14) $$\begin{array}{c} \left\{\begin{array}{c} W_{k}=W_{k}+1.0\\ W_{i}=W_{i},i\in \left(1,2,3\right)\setminus \left\{k\right\} \end{array}\right.ifL\left(new\_solution\right)>L\left(old\_solution\right)\\ \left\{\begin{array}{c} W_{k}=W_{k}\\ W_{i}=W_{i}+0.5,i\in \left(1,2,3\right)\setminus \left\{k\right\} \end{array}\right.ifL\left(new\_solution\right)\leq L\left(old\_solution\right) \end{array}$$

where L(solution) is the total value of successful observations of the solution (Formula (3)). In this formula, the following conditions apply:

1. If an operator *k* finds an improved solution (i.e., L(new_solution)>L(old_solution)), then $W_{k}$ is increased by 1.0, and other operator weights remain unchanged.
2. If the operator does not yield a better solution, then $W_{k}$ remains the same, and each other operator’s weight increases by 0.5.

Each neighborhood search uses a roulette wheel method to select operators, and the probabilities of the three operators being selected are $P_{1}$, $P_{2}$, and $P_{3}$. The calculation formula is as follows [24]:

(15) $$P_{j}=\frac{W_{j}}{\sum_{j=1}^{3}W_{j}},j=1,2,3$$

Among them, $W_{j}$ is the weight corresponding to operator *j*, and $\sum_{j=1}^{3}W_{j}$ is the sum of the weights of all operators.

#### 3.4.1. AVNS Algorithm Process

**Figure 10:**
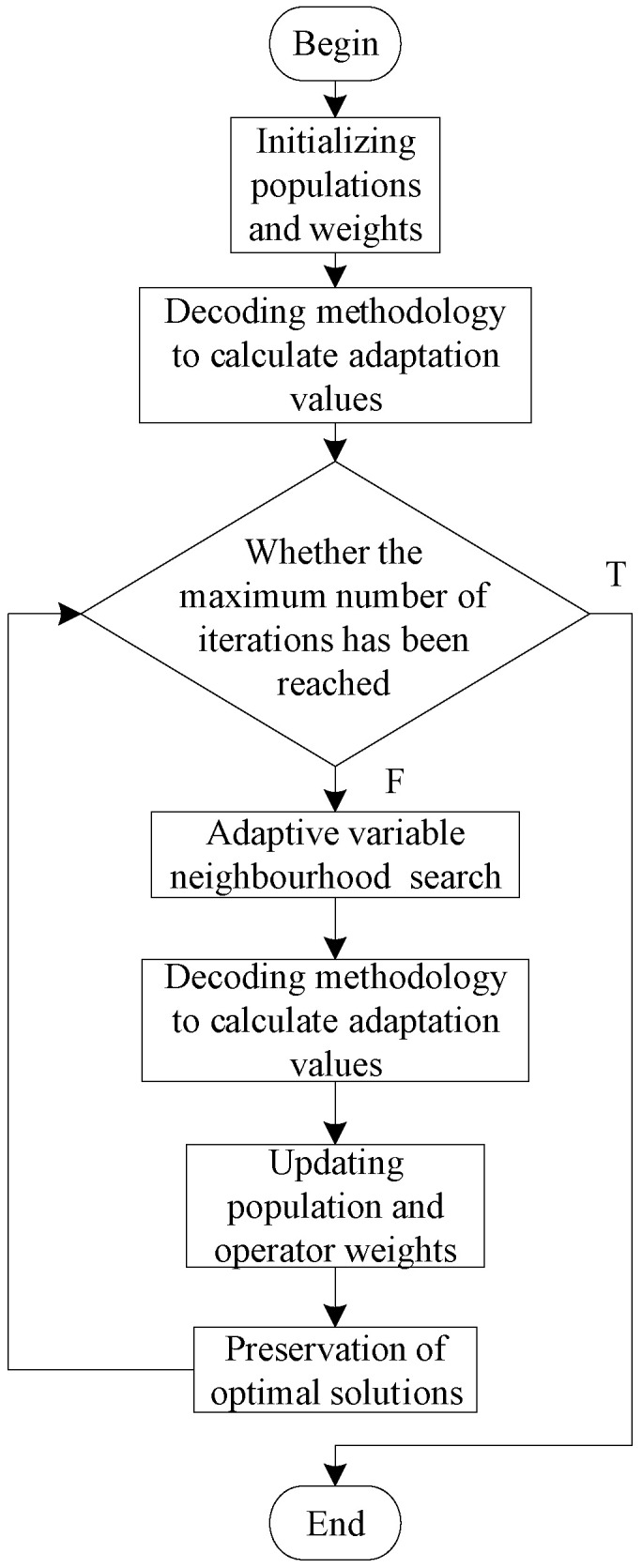
AVNS algorithm flowchart.

The flowchart of the AVNS in this paper is shown in Figure 10.

The algorithm flow is as follows:

1. Initialize weights and generate a certain number of initial solutions.
2. Total observed values are calculated using the decoding method proposed in Section 3.3.2.
3. Determine if the algorithm termination condition is met.
4. Select a roulette wheel operator for each individual in the population and search for neighborhoods using the selected operator.
5. Total observed values are calculated using the decoding method proposed in Section 3.3.2.
6. Update the population and save the optimal solution.
7. Repeat steps 3 to 6 until the termination condition is met and the algorithm terminates.

During the initialization process, we first set the initial weights of all three operators to 1.0. Subsequently, we generate initial solutions based on population size. When the algorithm generates an initial solution, it first forms a set using all the target numbers in the instance. The set obtained from the example in Table 1 is $\left\{12,13,16,29,58\right\}$. Subsequently, randomly sorting the target numbers in the set to generate a sequence of target numbers yields an initial solution, such as $\left[29,58,12,16,13\right]$.

The pseudo-code of the algorithm in this paper is shown in Algorithm 1.
**Algorithm 1** AVNS**Require:** Best solution**Ensure:** Target data, pop_size, telescope_number,gen, i=0 1: Initial_population() 2: Initialize_operator_weights() 3: **for** Each solution **do** 4:     Section 3.3.2 decoding_method() 5: **end for** 6: **while** i<gen **do** 7:     **for** Each solution **do** 8:         Adaptive_variable_neighborhood_search() 9:         Section 3.3.2 decoding_method() 10:     **end for** 11:     Update_population() 12:     Update_operator_weights() 13:     i+=1 14: **end while**

pop_size is the population size, telescope_number is the number of telescopes, and gen is the maximum number of iterations. Section 3.3.2’s decoding_method() is used to calculate the total observation value of each solution.

#### 3.4.2. Insert Operator

**Figure 11:**
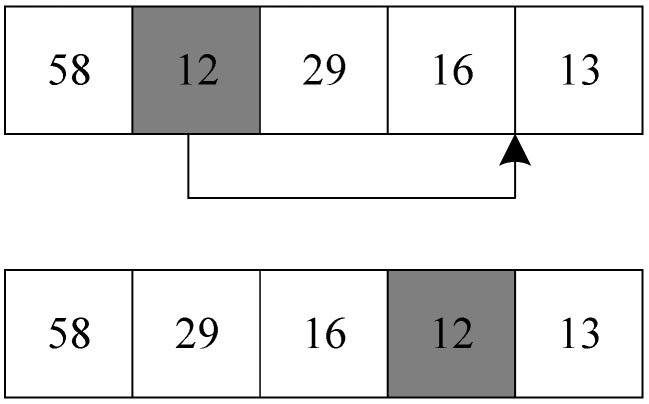
Insertion operator.

The insertion operator randomly selects a target number in the solution, removes the selected target number from its original position, and inserts it into other positions in the solution to generate a new neighborhood solution. The process of the insertion operator to generate a new neighborhood solution is shown in Figure 11.

Taking the solution in Section 3.3.1 as an example, the insertion operator selects target number 12 in the second position in the solution and inserts target 12 into target number 16 to produce a new solution.

#### 3.4.3. Commutative Operator

**Figure 12:**
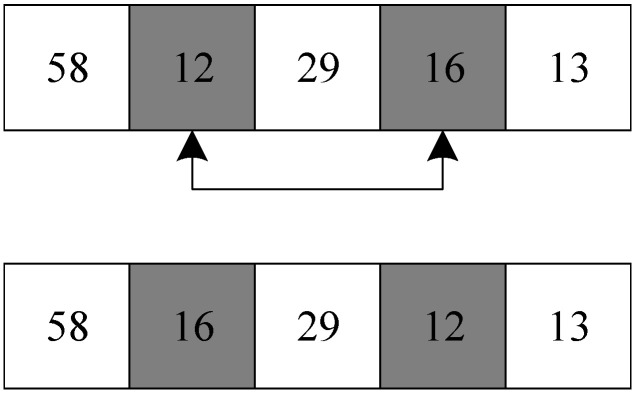
Commutative operator.

The commutative operator generates a new neighborhood solution by exchanging the target numbers at two different positions in the solution. The process of generating new neighborhood solutions through the commutative operator is shown in Figure 12.

Taking the solution in Section 3.3.1 as an example, the commutative operator swaps the positions of the target number 12 and the target number 16 in the solution, producing a new solution.

#### 3.4.4. Two-Opt Operator

**Figure 13:**
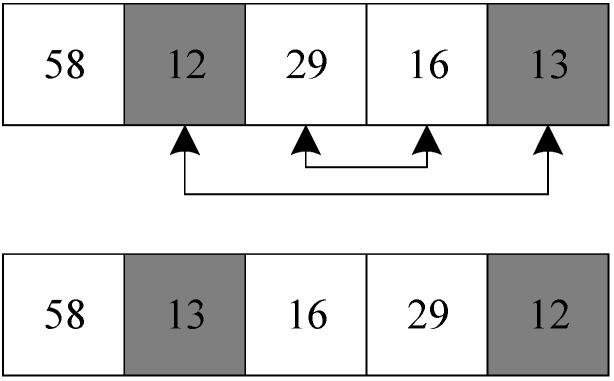
Two-opt Operator.

The two-opt operator selects two different positions in the solution and flips the sequence of target numbers between the two positions to generate a new neighborhood solution. The process of generating a new neighborhood solution by the two-opt operator is shown in Figure 13.

Taking the solution in Section 3.3.1 as an example, the two-opt operator selects the queue from target number 12 to target number 13 and inverts the target numbers to form a new solution.

#### 3.4.5. Complexity Analysis

We suppose that there are *m* telescopes and *n* space debris in the telescope observation of space debris scheduling problem, and the population size of the algorithm is *N*. The computational complexity of the total successful observation value is $O\left(nm\right)$. The time complexity of generating *N* random initial solutions is $O\left(Nn\right)$. Therefore, the time complexity of generating initial solutions and calculating the total successful observation value for each solution is $O\left(Nmn^{2}\right)$.

In the adaptive variable neighborhood search stage, the time complexity of all three operators is $O\left(mn^{2}\right)$, so the time complexity of this stage is $O\left(Nmn^{2}\right)$. Accordingly, the computation complexity of the AVNS is equal to $O\left(Nmn^{2}\right)$.

## 4. Experimental Studies

In order to verify the performance of the algorithm in this paper, the experiment used ten spatial target observation instances provided by relevant units, each containing about 9000 target observation information units. According to the problem’s difficulty, instances with more than 800 data points are considered large-scale problems, instances with 500–800 data points are considered medium-scale problems, and instances with less than 500 data points are considered small-scale problems. The computer’s operating system was Windows 11, the CPU was i7-12700h, and Python was the programming language. Due to the simple and efficient characteristic of the adaptive variable neighborhood search algorithm, the algorithm had only one parameter, POP_SIZE, and population size was set to 10. To obtain the algorithm’s source code in this article, please visit https://github.com/shixin63/AVNS (accessed on 10 November 2024).

In order to verify the validity of the decoding approach proposed in this paper, the decoding approach in this paper was compared with the greedy decoding approach. The greedy decoding method process is as follows:

1. Sort the data in ascending order according to when the target started to be observed.
2. Determine whether the target can be inserted into the end of the observation queue. If it meets the insertion conditions, insert the end of the observation queue.
3. If the target is inserted into the end of the observation queue of the current telescope, then the target number will be added to the set of observed queues; otherwise, continue to determine whether the target can be added to the end of the observation queue of the second telescope until the target is inserted into the end of the telescope queue or each telescope queue cannot be inserted into and the end of the insertion process is over.
4. Read the target data in the following data table and continue with steps 2 and 3 until all data have been executed through steps 2 and 3, ending the decoding.

We took the 2000 target observations from each of the ten instances, set the number of telescopes to two, and used greedy decoding and the decoding method proposed in this paper for the data in each instance, respectively. The experimental results are shown in Table 9.

The experimental results in Table 9 show that the decoding method proposed in this paper achieves significantly better decoding results than the greedy decoding method in all instances.

In order to verify the effectiveness of the problem decomposition strategy proposed in this article, an ablation experiment was conducted while ensuring that the algorithm’s operating environment and parameters remained unchanged. The experiment was divided into three situations: (1) Decompose the original problem into four sub-problems. (2) Decompose the original problem into two sub-problems. (3) Solve the original problem directly without using the problem decomposition strategy. Each case was calculated independently ten times on ten instances. The number of telescopes was set to two, each calculation instance took 1000 pieces of data, and the algorithm termination condition was to run for 50 generations. The experimental results are shown in Table 10. The time in the table represents the average time (in seconds) for the algorithm to run independently ten times on each instance from initialization to obtaining all observation plans.

The data in Table 10 show that when solving four sub-problems, the solution speed is the fastest, and the solution quality is higher than when directly solving the original problem, accounting for about 29% of the original problem-solving time. The best solution is achieved when solving two sub-problems, with a solution time of approximately 47% of the original problem. From this, it can be seen that the problem decomposition strategy proposed in this article can effectively improve the algorithm’s solving efficiency.

The experimental results show that, if each sub-problem can be solved in parallel, the algorithm’s execution time can be significantly shortened. However, as the problem is continually decomposed into smaller sub-problems, the reduction in solving time becomes progressively more minor, and some local optimality issues may arise. If parallel computing is to be used, after selecting the appropriate number of sub-problems, the communication between different sub-problem-solving processes must also be addressed. For example, when a target *n* is assigned to the observation queue in sub-problem 1, there is no need to add target *n* to the observation queue while solving other sub-problems. Some studies have explored communication methods in the parallel solving of scheduling problems but, so far, suitable methods for communication between sub-problem solutions in this paper have yet to be identified.

The algorithm in this article independently calculates ten times on ten instances, with the number of telescopes set to five. Each instance takes 2000 pieces of data. Due to the larger instance scale compared to the ablation experiments in Table 10, the original problem was divided into eight sub-problems on an instance with 2000 data points to speed up the algorithm’s solution. The termination condition of the algorithm was to run for 50 generations. The experimental results are shown in Table 11, where target number represents the number of observation targets included in 2000 data points, the average count represents the average number of observation targets after ten calculations, the average represents the average observation value in 10 calculations, and the maximum represents the maximum observation value obtained in 10 calculations.

From the data in Table 11, it can be seen that the algorithm in this paper can ensure that most of the targets are observed in the case of five telescopes and 2000 pieces of data, and only a tiny part of the targets that cannot be observed due to the observation time constraints are missed. In the problem-solving process, the replacement strategy of the decoding method can try to observe high-value targets first to ensure that high-value targets enter the observation queue to the maximum extent.

The effectiveness of the algorithm strategy proposed in the article has been demonstrated through previous experiments. We continued to compare the algorithm presented in this article with Genetic Algorithm (GA) [32,33] and Simulated Annexing Algorithm (SA) [34,35]. It is worth mentioning that the fewer dispatchable telescopes there are, the less observation queue space is available for the target, and the more complex the solution to the corresponding problem is. Let the number of telescopes be two; all algorithms are computed independently ten times on each of the ten instances, and 1000 data points are taken for each instance.

The algorithm in this article divided the problem into four sub-problems, with a population size of 10 and an iteration count of 50. GA used the POX crossover operator during the crossover process and the commutative operator during the mutation process. In each iteration, another solution was selected for each solution in the population based on the fitness roulette. Subsequently, the similarity between the observation queues of the two solutions was assessed. If they were dissimilar, crossover occurred; if they were similar, no crossover was performed. If a better solution was generated after crossover and added to the population, the population remained unchanged. In each round, a swapping operation was performed on each solution, where only the positions of two target numbers within the solution were exchanged, and only the better solution was accepted. The population size of GA was set to 10. The initial temperature of the SA was set to 100, with an initial temperature decrease rate of 0.98. The number of iterations for the isothermal process was set to 20, and the neighborhood search operator was selected as the swapping operator. The running time of GA and SA was the same as the CPU time required for 50 iterations of the algorithm in this article. Both comparison algorithms and AVNS used the decoding method proposed in this paper, and the experimental results are shown in Table 12 and Table 13.

As seen from the data in Table 12 and Table 13, the present algorithm ensures the scheduling of most of the targets into the observation queue, even in the more complex case of two telescopes. At the same time, the maximum value, average value, and average number of observed targets obtained by the proposed algorithm are superior to the comparison algorithms. AVNS uses three combined operators to have more robust local and global search capabilities than GA and SA. When the search falls into a local optimal state, it can adjust the weight to select the two-opt operator with a more extensive search range. When the operator needs local search, it can choose the insertion and commutative operators for search.

The fewer telescopes there are, the less observation time they provide, making it more difficult to obtain an observation plan with a higher total observation value. In order to demonstrate this phenomenon more intuitively, experiments were conducted with 10 telescopes. A total of 1000 data points were taken from each instance, and the algorithm independently calculated 10 times for each instance. The termination condition for the algorithm was that it stopped running after ten or five iterations without producing a better solution. The experimental results are shown in Table 14.

According to the experimental results in Table 14, it can be seen that, with ten telescopes compared to two telescopes, better solution results can be obtained with less solving time, and all targets in each instance are successfully observed. However, an excessive number of telescopes can cause some telescopes to remain idle for a long time, resulting in resource waste. Therefore, further research is needed to set the number of telescopes in the algorithm in future studies.

In summary, the performance of the scheduling methods and algorithms in this paper was tested by simulation experiments on 2000 data points taken in each of the ten arithmetic cases used in real work. The algorithm’s performance was also tested by taking 1000 data points in each example in the more complex case of two telescopes. The experimental results all show that the algorithms in this paper perform well in the data sets used in the actual work and can find a better solution within an acceptable time.

## 5. Conclusions

In this paper, a telescope scheduling model was established according to the actual needs of space observation and telescope array scheduling. A coding method based on target priority was proposed relying on the actual data, and a decoding method matching the coding method was designed. In order to solve the problem that the algorithm has difficulty searching on huge dimensional arithmetic cases, a problem decomposition strategy was designed. On this basis, an adaptive variable neighborhood search algorithm was used to solve the telescope scheduling problem, and the effectiveness of the algorithm proposed in this paper was verified.

The combination of coding and decoding methods proposed in this paper effectively reduced the size of the solution space and facilitated the algorithm solving the problem efficiently. Three insertion methods and one substitution method for the target to enter the telescope observation queue were proposed, and the effectiveness was proved in simulation experiments using actual data. The proposed problem decomposition method can split a problem into several small-scale sub-problems, and the split sub-problems can be solved separately and combined to shorten the problem-solving time. Based on the above strategy, excellent results have been achieved in solving the telescope scheduling problem using the adaptive variable neighborhood search algorithm.

The effectiveness of the model, coding method, decoding method, and problem decomposition strategy established in this paper were verified by simulation experiments using actual data, which proved that the algorithm used in this paper can meet the requirements of telescope observation scheduling. The problem decomposition strategy proposed in this article abandoned some observable periods of the target when decomposing the original problem into sub-problems, as they were less than $T_{n}$ after decomposition. These periods are observable and will be sought to solve this problem in future research. The next step is to conduct more in-depth research on the coding and decoding methods to further improve the search speed of the algorithm in such a large problem dimension as 9000 data points. The application of deep reinforcement learning methods in combinatorial optimization problems is a hot topic in current research, and future research work can explore the use of deep reinforcement learning to solve telescope scheduling problems [36,37,38,39]. In practice, in addition to maximizing the scientific value of observations, the scheduling of telescope observations usually considers factors such as operating costs [11,15]. Therefore, studying multi-objective algorithms for the telescope observation scheduling problem is also of great value [40,41,42,43,44,45,46].

## Figures and Tables

**Table 1:** An example of target data.

Target Number	Target Level	Initial Time	Deadline
n	$l_{n}$	$O_{n}$	$D_{n}$
12	3	76,925	77,025
13	3	77,015	77,115
16	1	75,833	76,121
16	1	76,609	77,109
16	1	84,585	85,345
29	1	106,755	106,921
29	1	112,615	113,022
58	1	76,925	77,020

**Table 2:** Sorted target observation data.

Target Number	Target Level	Initial Time	Deadline
n	$l_{n}$	$O_{n}$	$D_{n}$
2378	1	77,539	79,309
6848	9	77,562	78,349
2377	1	77,568	77,876
4259	8	77,614	78,082
4617	7	79,255	79,448
6266	1	79,908	80,176
2121	3	79,994	80,267

**Table 3:** First set of target observation data.

Target Number	Target Level	Initial Time	Deadline
n	$l_{n}$	$O_{n}$	$D_{n}$
2378	1	77,539	78,903
6848	9	77,562	78,349
2377	1	77,568	77,876
4259	8	77,614	78,082

**Table 4:** Second set of target observation data.

Target Number	Target Level	Initial Time	Deadline
n	$l_{n}$	$O_{n}$	$D_{n}$
2378	1	78,903	79,309
4617	7	79,255	79,448
6266	1	79,908	80,176
2121	3	79,994	80,267

**Table 5:** The observation plan obtained from the original problem.

Target Number	Target Level	Start Time	End Time
n	$l_{n}$	$S_{n}$	$C_{n}$
2378	1	77,539	77,629
6848	9	77,562	77,652
2377	1	77,652	77,742
4259	8	77,742	77,832
4617	7	79,255	79,345
6266	1	79,908	79,998
2121	3	79,998	80,088

**Table 6:** Prioritized data table.

Target Number	Target Level	Initial Time	Deadline
n	$l_{n}$	$O_{n}$	$D_{n}$
58	1	76,925	77,020
12	3	76,925	77,025
29	1	106,755	106,921
29	1	112,615	113,022
16	1	75,833	76,121
16	1	76,609	77,109
16	1	84,585	85,345
13	3	77,015	77,115

**Table 7:** Observation queue after insertion.

Target Number	Target Level	Initial Time	Deadline
n	$l_{n}$	$S_{n}$	$C_{n}$
16	1	75,833	75,923
58	1	76,925	77,015
13	3	77,015	77,105
29	1	106,755	106,845

**Table 8:** Replaced observation queue.

Target Number	Target Level	Initial Time	Deadline
n	$l_{n}$	$S_{n}$	$C_{n}$
16	1	75,833	75,923
12	3	76,925	77,015
13	3	77,015	77,105
29	1	106,755	106,845

**Table 9:** Comparison of decoding methods.

Instance	Total Successful Observation Value	
	Greedy Decoding Method	Decoding Method of This Article
1	1148	1366
2	1114	1372
3	1158	1368
4	1143	1383
5	1161	1407
6	1159	1418
7	1175	1405
8	1157	1375
9	1201	1410
10	1200	1374
Average	1161.6	1387.8

**Table 10:** Ablation experiment.

Instance	4 Sub-Problems		2 Sub-Problems		Original Problem	
	Time	Value	Time	Value	Time	Value
1	2083.8	794	3028.7	801.3	7663	763
2	1847.3	767.9	2938.6	776.4	7243.5	741.4
3	2020.1	765.5	3105.3	767.7	6578.4	732.6
4	1976.6	774.5	3218	781.1	7028.5	740.4
5	2044	786.9	3443.6	798.3	6091.2	751
6	2085.2	768.1	3394	774.3	6690.5	735.3
7	1943.1	781.8	3352.2	787.3	6575.6	745.9
8	1890.1	757.1	3287.1	766.3	6766.6	723.3
9	1890.1	752	3200.6	765.4	6873.6	720.4
10	1995.9	727.5	3042.3	736	6352.3	697.4
Average	1977.62	767.5	3201.04	775.4	6786.32	735.1

**Table 11:** Experimental results.

Instance	Target Number	Maximum	Average	Std. Deviation	Avg. Count
1	1175	1710	1700.6	4.78	1130.3
2	1161	1686	1680	3.46	1122
3	1171	1688	1680.3	3.88	1125.3
4	1169	1693	1685.6	4.10	1129.9
5	1195	1749	1745	3.70	1151
6	1196	1756	1744.4	3.89	1150.3
7	1196	1749	1752.4	2.50	1150.4
8	1191	1740	1737	2.27	1155.9
9	1189	1756	1752.4	3.96	1145.3
10	1151	1686	1680.6	2.56	1103.6

**Table 12:** Comparative experiment.

Instance	Target Number	Time	AVNS				GA			
			Maximum	Average	Std. Deviation	Avg. Count	Maximum	Average	Std. Deviation	Avg. Count
1	592	2118.5	814	794	11.80	536.5	765	762.7	6.24	518.6
2	573	1905	788	767.9	8.12	525.625	736	731.4	5.71	496.4
3	578	2213.3	778	765.5	8.65	522.75	730	728.3	4.74	500
4	590	1999.7	778	774.5	3.00	539	737	733.7	9.21	504.4
5	601	2021.3	791	786.9	3.02	538.375	750	747.1	5.29	499.9
6	592	1971.4	770	768.19	1.96	538.625	733	729.9	5.83	502
7	591	2000.6	784	781.8	2.54	539.5	744	742.4	5.39	502.9
8	581	2013.7	761	757.1	2.42	558.125	721	719	8.70	497.1
9	582	2068.9	758	752	3.32	528.5	720	716.3	5.14	494
10	569	2007.6	730	727.5	2.55	519.5	698	691.9	5.82	489.4
Average	584.9	2032	775.2	767.539	4.74	534.65	733.4	730.3	6.21	500.5

**Table 13:** Comparative experiment.

Instance	Target Number	Time	AVNS				SA			
			Maximum	Average	Std. Deviation	Avg. Count	Maximum	Average	Std. Deviation	Avg. Count
1	592	2118.5	814	794	11.80	536.5	779	771.8	5.42	529.5
2	573	1905	788	767.9	8.12	525.625	746	733.3	6.28	504.3
3	578	2213.3	778	765.5	8.65	522.75	748	740.4	6.92	513.8
4	590	1999.7	778	774.5	3.00	539	755	744.6	5.28	518.4
5	601	2021.3	791	786.9	3.02	538.375	771	763.4	5.72	519.2
6	592	1971.4	770	768.19	1.96	538.625	753	729.9	6.72	516.2
7	591	2000.6	784	781.8	2.54	539.5	744	740	4.76	517
8	581	2013.7	761	757.1	2.42	558.125	758	752.8	5.27	509.2
9	582	2068.9	758	752	3.32	528.5	741	730	7.47	505
10	569	2007.6	730	727.5	2.55	519.5	712	706.3	3.69	503.3
Average	584.9	2032	775.2	767.539	4.74	534.65	750.5	740.97	5.75	513.59

**Table 14:** 10 Telescope experiment results.

Instance	Target Number	Maximum	Average	Avg. Count	Avg. Time
1	592	835	835	592	145.4
2	573	806	806	573	189.6
3	578	807	807	578	128.3
4	590	819	819	590	199.7
5	601	848	848	601	221
6	592	819	819	592	204.2
7	591	830	830	591	156.6
8	581	804	804	581	148
9	582	805	805	582	209.4
10	569	774	774	569	180.6
Average	584.9	814.7	814.7	584.9	178.3

## Data Availability

The data that support the findings of this study are available from the corresponding author upon request. There are no restrictions on data availability.
